# The Book World of Medicine and Science

**Published:** 1894-03-03

**Authors:** 


### The Book World of Medicine and Science.

### BOOK REVIEWS.

Headache and Other Morbid Cephalic Sensations. By Harry Campbell, M.D., B.S. (Lond.), Physician to the North-West London Hospital, Member of the Royal College of Physicians. (London: H. K. Lewis, 136, Gower Street. Price 12s. 6d.)

Dr. Campbell's volume says all that is to be said on a subject that is not so trivial as it looks. "Only a headache" may be the indication of the onset of many serious diseases; it is a concomitant of almost all, and in the kind of headache from which a patient suffers may be found a guide to the flaw in the organism. Dr. Campbell deals with every possible variety of headache, its possible cause and scientific treatment, with a detail which may possibly sometimes be found confusing, and may lead young practitioners (after the manner of Wendell Holmes and Benjamin Franklin in the matter of the bruise on the patient's brow) to assign the most remote and serious causes to the simple headache of constipation or fatigue. To a judicious reader, however, it will prove a useful and suggestive work, and will give many hints as to the value of the apparently minor symptoms of ill-health.

Essays on Rural Hygiene. By Dr. Vivian Poore. (Published by Longmans, Green, and Co.; price, 6s. 6d.)

This volume of collected papers on sanitary matters is a valuable addition to the literature that already exists on this most important subject, and the value of these essays is certainly increased by their popular character and by the total absence of all technicalities. The writer does not claim to set forth anything new, but merely repeats in clear and forcible language facts which are universally acknowledged, but are very rarely even now carried into practice. Since the essay on "Concentration of Population in Cities" was written, more than a year ago, this subject has attracted a good deal of attention, and has been widely discussed, but there has not been much practical result from it as yet. Dr. Poore calls attention to the dangers of over-concentration of population in cities, the craze for money-making being fostered by tenement houses, whereby the largest possible number of people is crowded into the smallest possible space. These tenement houses have been largely adopted in New York and Paris, and in both cases with disastrous effects and a greatly-increased death-rate. Nor, according to Dr. Poore, is the harm limited to the material welfare of the population of London, but "the filthiness of the air of London is a not unimportant factor in causing the moral degradation in which not a few of the inhabitants are sunk," and is one of the means by which London justifies the remark that it is "a city where an abundance of second-rate health exists." In the chapters entitled "The Living Earth," "Practical Details," "Personal Experiences," and "Burial," the author sets forth the great advantage of what he calls "living" earth, *i.e.*, that within about a foot of the surface, in contradistinction to the deep soil, as the best, because the most natural method of disposing of all putrescible material.

Some of the most interesting chapters in this volume are those in which the author gives his experiences in a suburban garden fertilized by house refuse and excremental matter. Few people are both enthusiastic gardeners and thorough and scientific sanitarians, but in Dr. Poore these rare qualities are combined, and hence the value of his experiments. The method he adopted was the daily removal of all refuse which was buried—not too deep, so as to reach the "dead" earth, but in the "living" earth, where the process of nitrification goes on rapidly without the air being polluted; all ammoniacal smells should warn the gardener that valuable material is being lost. After ten years' experience of this system the author says, "The only fault which I have to find with it is that the crops have a tendency to be gross and too luxuriant."

As regards large towns, the author would like to see the same system adopted, and the material applied in a similar manner to the soil of our large public gardens. This would be a difficult matter, requiring the intelligent co-operation of a large number of people who know little and care less about sanitary matters, and we fear it will be long before such a desirable change can be effected. This volume closes with the "Story of Bremontier," the engineer who at the beginning of this century originated the idea of reclaiming the "Landes," and by judicious planting has converted them from a pestilential region to a healthy prosperous district, carrying out his almost herculean work in spite of much opposition. We heartily commend this excellent volume to the perusal of all, and feel sure that no one will read it without deriving useful and eminently practical hints from it.

Aspects of Modern Oxford. By "A Mere Don." (London: Seeley and Co. 1894.)

The writer of this book has shown himself in his work to be something more than the "Mere Don" that he describes himself; indeed, the author has placed himself in a position hitherto unattained (though frequently attempted) of being a faithful biographer of modern Oxford. For to describe life at Oxford is no easy thing, it is at once so complex and so many sided that much is required at the hands of the historian who would tell its story. No place of education has undergone greater changes, both in the spirit and the detail of its existence, and to understand Oxford as it is one must understand Oxford as it was.

The brilliantly-coloured pictures of the undergraduate life of fiction and the drama may once have been a living reality, but as "A Mere Don" remarks, they exist no more. "The town and gown rows which used to provide so attractive a picture for the novelist—where the hero used to stand pale and determined, defying a crowd of infuriated bargemen—are extinct and forgotten these last ten years . . . when the anarchial element is now loose it seems to prefer the interiors of colleges." But the "man" of modern times as opposed to his more youthful predecessor is a far less picturesque creature. "He is on the whole," we are told on the authority of the book we are discussing, "inexpensive in his habits, as it is now the fashion to be poor. . . ." In the days when the streets of pre-collegiate Oxford were cleared by the Proctor with a pole-axe the mediæval student existed for the University; modern proctorial discipline is a more parlous matter now, and the University it is which exists for the student. The mediæval student came to Oxford primarily for the love of learning something; the student *fin de siècle* is one of the most labyrinthine parts of a complex civilisation; other and varied motives they are which prompt the academic career on the part of the nineteenth century undergraduate. Altogether they are a motley crew, and it is not the least achievement of the University our author points out to us, "That she does somehow or other manage to impress a certain stamp on so many different kinds of metal."

When we close this book, the outcome of a scholarly mind sparkling throughout with humour at once witty and genial, we are conscious of a deep sense of regret that "A Mere Don" does not initiate us yet further into the interesting and mysterious intricacies of life at Oxford. If space permitted we should like to say more of the illustrations with which the work abounds, and which are cleverly chosen in bringing to our mind not alone the scholastic element at the great city of learning, but that other side—the happy holiday side of life at Alma Mater.

